# The dynamics of reading complex words: evidence from steady-state visual evoked potentials

**DOI:** 10.1038/s41598-021-95292-0

**Published:** 2021-08-05

**Authors:** Elisabeth Beyersmann, Veronica Montani, Johannes C. Ziegler, Jonathan Grainger, Ivilin Peev Stoianov

**Affiliations:** 1 Department of Cognitive Science and Macquarie Centre for Reading, Macquarie University, Australian Hearing Hub, 16 University Avenue, Sydney, NSW 2109 Australia; 2 Department of Neurosciences, Biomedicine, and Movement Sciences, University of Verona, Verona, Italy; 3 Laboratoire de Psychologie Cognitive, Aix-Marseille University and Centre National de La Recherche Scientifique, Marseille, France; 4 Institute of Cognitive Sciences and Technologies, National Research Council, Padova, Italy

**Keywords:** Neuroscience, Psychology

## Abstract

The present study used steady-state visual evoked potentials (SSVEPs) to examine the spatio-temporal dynamics of reading morphologically complex words and test the neurophysiological activation pattern elicited by stems and suffixes. Three different types of target words were presented to proficient readers in a delayed naming task: truly suffixed words (e.g., *farmer*), pseudo-suffixed words (e.g., *corner*), and non-suffixed words (e.g., *cashew*). Embedded stems and affixes were flickered at two different frequencies (18.75 Hz and 12.50 Hz, respectively). The stem data revealed an earlier SSVEP peak in the truly suffixed and pseudo-suffixed conditions compared to the non-suffixed condition, thus providing evidence for the form-based activation of embedded stems during reading. The suffix data also showed a dissociation in the SSVEP response between suffixes and non-suffixes with an additional activation boost for truly suffixed words. The observed differences are discussed in the context of current models of complex word recognition.

One of the keys to becoming a skilled reader is the ability to rapidly process complex words like *paint-er, paint-ing,* and *paint-brush*. Complex words consist of multiple orthographic building blocks (“morphemes”) which contribute to the overall meaning of the word (e.g., *a painter is someone who paints*). Since the early work of Taft and Forster^1^, visual word recognition research has attempted to explain the phenomenon of complex word identification and generated debate as to what the exact underlying cognitive mechanism are. Several competing theoretical strands have formed over the years, which disagree over how exactly skilled readers gain access to the internal structure of words with multiple morphemes, and to which extent morphological processing is governed by semantics^2,3^. However, what most recent theoretical accounts do agree on is that skilled readers are experts at rapidly and automatically extracting morphological information from print^3–6^.

Given the speed and proficiency by which morphological information is processed in visual word recognition, it has been an experimental challenge to track down and record the time-course and mechanisms that lie behind it. One technique that has been able to shed light onto mechanisms of morpheme identification is the masked primed lexical decision paradigm^7^. In a typical masked priming experiment, target words are preceded by a masked prime, which is presented so briefly (40–60 ms) that participants are not aware of its existence. Yet, facilitatory or inhibitory influences of the prime onto the target word can be measured, thus providing insights into the early, automatic stages of visual word recognition. One of the key breakthroughs in the masked morphological priming literature^4,5,8^ has been the finding that not only words with a true morphological structure prime their embedded stems (*farmer-farm*), but also words with a morphological pseudo-structure (*corner-corn*). Crucially, no such priming is seen with non-affixed words (*cashew-cash*), which rules out the possibility that the observed priming effects are simply due to the orthographic overlap between the prime and the target. This by now widely replicated result^3^ provides evidence for a fast-acting segmentation mechanism that skilled readers use to identify morphological structure, rapidly and independently of semantics.

In more recent years, results from behavioural priming experiments have been complemented by neuro-imaging data and provided a more fine-tuned perspective onto the temporal dynamics of the early reading stages. Studies using a combination of masked priming and high-temporal resolution recordings of event-related brain potentials (ERPs)^9–17^ have shown no difference between true morphological and pseudo-morphological priming in earlier time windows. These ERP results thus demonstrate that the initial stages of complex word recognition are based on a purely structural form or morphological analysis, whereas the later processing stages are more likely to be influenced by semantics^18^.

The question of how morphologically complex words are decomposed into morphemes has also been investigated by studying neural responses to a simple (unprimed) lexical decision task using MEG. Several MEG studies have shown that form-based morphological decomposition takes place at around 170 ms post-stimulus onset^19–22^. While some studies reported a right-lateral effect of morphological complexity^19^, others have located the M170 effect in the left fusiform gyrus^21,22^, i.e. the visual word form area, where special sensitivity to letter strings—as opposed to other visually similar stimuli—has consistently been localized^23,24^. These MEG findings suggests that the M170 represents an early, pre-lexical index for the extraction of morphemic forms embedded in complex words.

Several theories of morphological processing have picked up the idea that there is an early processing stage that is mostly driven by form, and then a later, more central processing level where semantics is more heavily involved^3,4,25^. These models were based on the first observations of masked versus unmasked morphological priming^26,27^ and then fully formalised later^25,28–32^. However, although the review of the literature clearly shows that skilled readers are experts at rapidly identifying morphological structure from print, it is still uncertain how exactly the reading system achieves the rapid segmentation of letter strings into morphemic subunits.

The more recent “word and affix” model by Grainger and Beyersmann^33,34^ captures the idea of early morphological segmentation in the form of morpho-orthographic full decomposition. The model predicts that embedded words (e.g., *corn* in *corner*) and suffixes (e.g., *er* in *corner*) are mapped onto existing representations in the orthographic lexicon. The active stem and affix representations then undergo the morpho-orthographic full decomposition check, captured in the links between the orthographic input and the orthographic lexicon, to analyse whether or not the input letter string can be exhaustively decomposed into morphemes. If successful, as in the case of *farm* + *er* and *corn* + *er*, this generates a boost in activation to the embedded stem (*farm, corn*). Finally, the orthographic form representations are mapped onto a layer of semantic representations^33,34^. In this regard, the model resembles earlier form-then-meaning theories of morphological processing^18,29,32^, suggesting that the early stages of morphological analyses operate independently of semantics. However, the word and affix model makes the specific, additional prediction that the reading system differentiates between stems and affixes during the early stages of complex word reading. While embedded stems can occur in various positions (e.g., *book* in *textbook* or *bookshelf*), affixes have clear positional constraints (i.e., suffixes occur at the end, prefixes at the beginning). Moreover, letter position encoding is flexible for embedded words to allow for the activation of orthographically underspecified stems (e.g., *ador* in *adorable*) and orthographically similar words. In contrast, letter position encoding needs to be specific for the process of affix activation^35^, to ensure that affixes are only identified when the letters of the input provide a precise orthographic match (e.g., the precise match of -*ment* in *development* compared to the non-consecutive letter string in *circumvent*). The word and affix model therefore predicts that the neurophysiological activation pattern of stem and affix forms should be clearly dissociable.

Prior neurophysiological research, such as the above-mentioned studies, have focussed on the relationships between complex words and their embedded stems (e.g., by testing the influence of whole words on their embedded stems in masked priming). Only few studies have directly tapped into the neural activation pattern associated with stem and affix representations during visual word recognition. One notable study by Solomyak and Marantz^20^ used MEG to test lexical decision responses to morphologically complex words, and found that the early M170 component was significantly modulated by “morphological affix frequency” (the frequency of the affix as a morphemic unit), but not “orthographic affix frequency” (the frequency of the affix appearing at the end of any word). This research provides some initial evidence for the idea that affixes are associated with clearly distinct neurophysiological activation pattern. The goal of the present study was to use an entirely novel approach to investigate electrophysiological responses to morphemic form units during reading and directly tag stems and affixes by using steady-state visual evoked potential (SSVEP).

SSVEPs are electrophysiological responses of the cortex to flickering visual stimuli^36^ that presumably originate from the entrainment of brain oscillations^37,38^ through phase alignment^39,40^. They reflect the reorganization of the neural activity in response to the visual stimulation, and can be modulated by various cognitive tasks^41,42^. SSVEPs generated by flickering written words and pseudowords are sensitive to fundamental effects of reading aloud^43^ and to the effect of syllable processing^44^, by virtue of tagging the specific units using different stimulation frequencies simultaneously (i.e., frequency tagging). The analysis of the frequency bins corresponding to each stimulation frequency allows to extract the evoked responses originated from the population of cells that are selectively entrained by each stimulus^41^, and therefore, distinguish the responses associated with a particular segment of the word.

Following earlier work^44^, we adopted a delayed naming task in which the target words were split into two segments, each of which flickering at a distinct frequency. The first segment matched the stem of the word (e.g., *poche* [engl. *pocket*] in *pochette* [engl. *little pocket*]), and the second segment matched the remaining part of the word (e.g., the affix -*ette* in *pochette*). The goal was to dissociate participants’ EEG responses to embedded stems and affixes, by using two distinct frequencies. Since SSVEPs can be used to measure specific neural responses to embedded reading units, they offer a unique avenue to investigate neurophysiological activation pattern to affixes (i.e., “bound morphemes” that do not naturally occur in isolation).

The central aim of our study was to test a prediction of the word and affix model^33,34^ concerning the strength of morpheme activation in the mental lexicon. The model predicts strong, uninhibited activation of stems and affixes embedded in words with a true morphological structure (*farm* and *er* in *farmer*) as well as pseudo-stems and pseudo-suffixes embedded in words with a pseudo-morphological structure (*corn* and *er* in *corner*). In contrast, the activation of words embedded in non-affixed words is hypothesised to be hindered by inhibitory connections between the embedded word (*cash*) and the whole word (*cashew*) at level of the orthographic lexicon^45^. Moreover, non-morphemic endings like *ew* in *cashew* are not associated with any existing affix representations and should therefore be neurophysiologically clearly distinguishable from real affixes. To test this prediction, we included three different types of target words: truly suffixed words (*farmer*), pseudo-suffixed words (*corner*), and non-suffixed words (*cashew*).

The neural dynamics of morpheme activation were examined by analysing the temporal modulation of SSVEP power associated with the three types of words, for stems and suffixes separately. SSVEP power represents the amount of activity in the selected frequency bin in response to a specific stimulus^46^. It reflects the ability of the neural network to synchronize to the flickering event, which can be taken as an index of network efficiency^47,48^. Accordingly, the latency of the response reflects the speed at which the specific neural assembly responds to the flickering stimulus, while the magnitude of the response broadly reflects the size of the recruited network^46,48^. To estimate the modulation of the latency and magnitude of SSVEP responses associated with morphological processing, we approximated the time course of the responses for each condition using polynomial interpolation (see “Methods” for details). Shorter latencies and/or larger SSVEP magnitudes were expected for embedded (pseudo)morphemic sub-units, for which we predicted stronger and uninhibited activation compared to the non-morphemic letter sequences of the non-suffixed condition.

## Results

To inspect the time course of the effect of morphological decomposition on visual word recognition, the SNR of the SSVEP response from 200 to 1000 ms after word onset was subjected to mixed-effect multiple regression models^49^. To avoid overspecification, and following Mirman’s recommendations^50^, the choice of the specific order of polynomials was guided by a preliminary visual inspection. From the visual inspection, we estimated that the trajectories of the SSVEP responses to stems were characterized by up to two turning points, and therefore, could be modelled with third-order (cubic) polynomials, while the trajectories for suffixes were characterized by one turning point, and therefore, could be modelled with two-order (quadratic) polynomials. The final linear mixed effects model included the polynomial terms, Morpheme Status (TS, PS, NS), and the interaction between each polynomial term and Morpheme Status as fixed effects, plus by-subject random intercepts.

*P*-values were corrected using the Benjamini and Hochberg^51^ method to control for multiple comparisons. Statistical analyses were performed using lme4 package^52^, and lmerTest package^53^, while data visualization was realized using ggplot2 package^54^ and sjPlot package^55^, in the R environment^56^. The stem data (18.75 Hz) and the suffix data (12.50 Hz) were analysed separately. In addition, inter-modulation (IM) analyses were carried out to capture the interaction between the neural responses to stems and suffixes in the current data. The truly suffixed (TS) condition was treated as the reference (baseline) and relative parameters estimated for the pseudo (PS) and the non-suffixed (NS) condition, and then the pseudo suffixed condition was treated as the reference and relative parameters estimated for the non-suffixed condition (i.e., dummy coding).

### Results stems (18.75 Hz)

**Figure 1:**
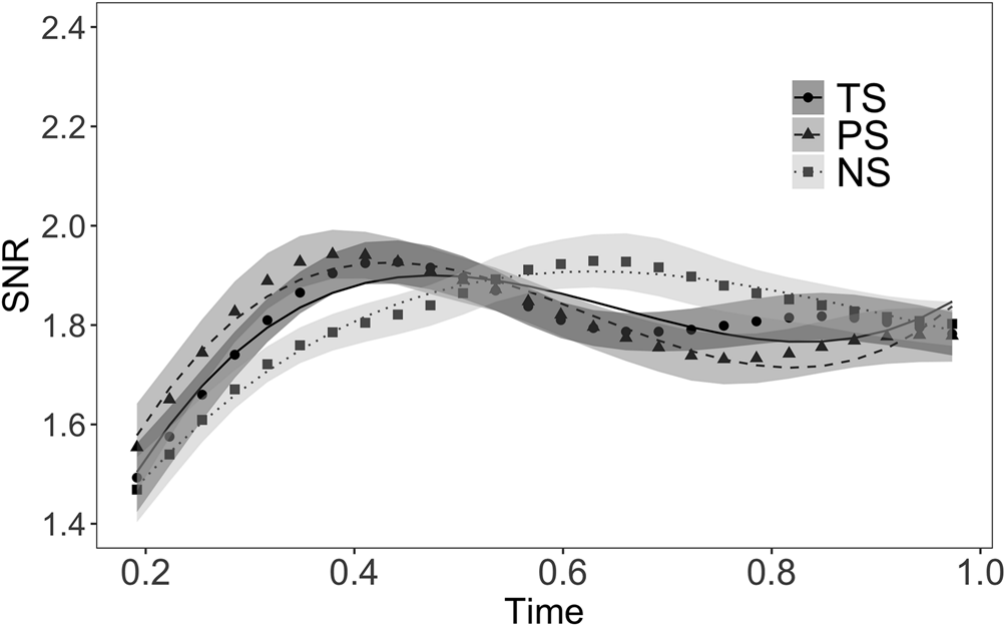
Effect of morpheme status for stems. The SSVEP time course from RESS component optimized for 18.75 Hz is shown. Symbols represent EEG data; shadows represent standard error of the means (SEMs) adjusted to correctly reflect the variance in the within-subject design (Morey, 2008); lines represent growth curve analysis model fit. TS = Truly suffixed, PS = Pseudo-suffixed, NS = Non-suffixed. The figure was created using R^56^ (http://www.R-project.org) package ggplot2 (https://cran.r-project.org/web/packages/ggplot2/index.html).

The effect of Morpheme Status, along with the growth curve analysis model fit, in the time interval 200–1000 ms, is shown in Fig. 1, and the final model parameters in Table 1. The b-coefficient represents the adjustment with respect to the reference level.

**Table 1:** Final model parameters for stems.

Predictors	Estimates	Std. Error	Statistic	*p* adjusted
**SNR**				
Intercept (TS)	1.80	0.11	16.70	< 0.001
Linear	0.14	0.05	2.89	0.006
Quadratic	− 0.32	0.05	− 6.69	< 0.001
Cubic	0.31	0.05	6.41	< 0.001
PS	0.00	0.01	0.14	0.891
NS	0.01	0.01	0.43	0.749
Linear * PS	− 0.21	0.07	− 3.10	0.003
Linear * NS	0.24	0.07	3.51	0.001
Quadratic * PS	0.08	0.07	1.14	0.322
Quadratic * NS	− 0.13	0.07	− 1.86	0.093
Cubic * PS	0.07	0.07	0.97	0.390
Cubic * NS	− 0.22	0.07	− 3.31	0.002
**Random effects**				
σ2	0.04			
τ00 subj	0.20			
ICC	0.83			
N subj	17			
Observations	1326			
Marginal R^2^/conditional R^2^	0.040/0.841			

Factors were dummy coded with truly suffixed as reference level, using polynomial interpolation. The *b*-coefficient (estimates) represents the adjustment with respect to the reference level. *TS* truly suffixed, *PS* pseudo-suffixed, *NS* non-suffixed.

#### Effect of morpheme status

The comparison between the TS and NS conditions (solid vs. dotted line, Fig. 1) revealed that there was no significant effect on the intercept (*b* = 0.01, *t* = 0.43, *p* = 0.750), indicating that, on the average, the magnitude of the response was similar for TS and NS. However, there was a significant effect on the linear term (*b* = 0.24, *t* = 3.51, *p* < 0.001), with a positive β, indicating that the SNR response increased later in the NS compared to the TS condition. There was no significant effect on the quadratic term (*b* = − 0.13, *t* = − 1.86, *p* = 0.09), however, there was a significant effect on the cubic term (*b* = − 0.22, *t* = − 3.31, *p* < 0.001), with a negative β, indicating less steep peaks for NSs compared to TSs, and therefore, reflecting, in particular, a faster initial increase for TSs compared to NSs.

The comparison between the TS and PS conditions (solid vs. dashed line, Fig. 1) revealed no significant effect on the intercept (*b* = 0, *t* = 0.14, *p* = 0.89), suggesting that the average response magnitude was comparable. There was only a significant effect on the linear term (*b* = − 0.21, *t* = − 3.1, *p* < 0.001), with a negative β, indicating that the overall increase was smaller in the PS than in the TS condition. Neither the quadratic term (*b* = 0.08, *t* = 1.14, *p* = 0.32) nor the cubic term (*b* = 0.07, *t* = 0.97, *p* = 0.39) were significant, showing that the slopes of two trajectories were similar.

The comparison between the PS and NS conditions (dashed vs. dotted line, Fig. 1) revealed no significant effect on the intercept (*b* = 0, *t* = 0.29, *p* = 0.83), indicating that the average response magnitude was comparable. There was a significant effect on the linear term (*b* = 0.45, *t* = 6.62, *p* < 0.001), with a positive β, showing a larger SNR increase in the NS condition compared to the PS condition. There was a significant effect on the quadratic term (*b* = − 0.2, *t* = − 3, *p* < 0.001), with a negative β, indicating a steeper (quadratic) curve in the NS compared to the PS condition. However, there was also a significant effect on the cubic term (*b* = − 0.29, *t* = − 4.28, *p* < 0.001), with a negative β, indicating rather the opposite, that is, a flatter (cubic) slope in the NS compared to the PS condition.

### Results suffixes (12.50 Hz)

**Figure 2:**
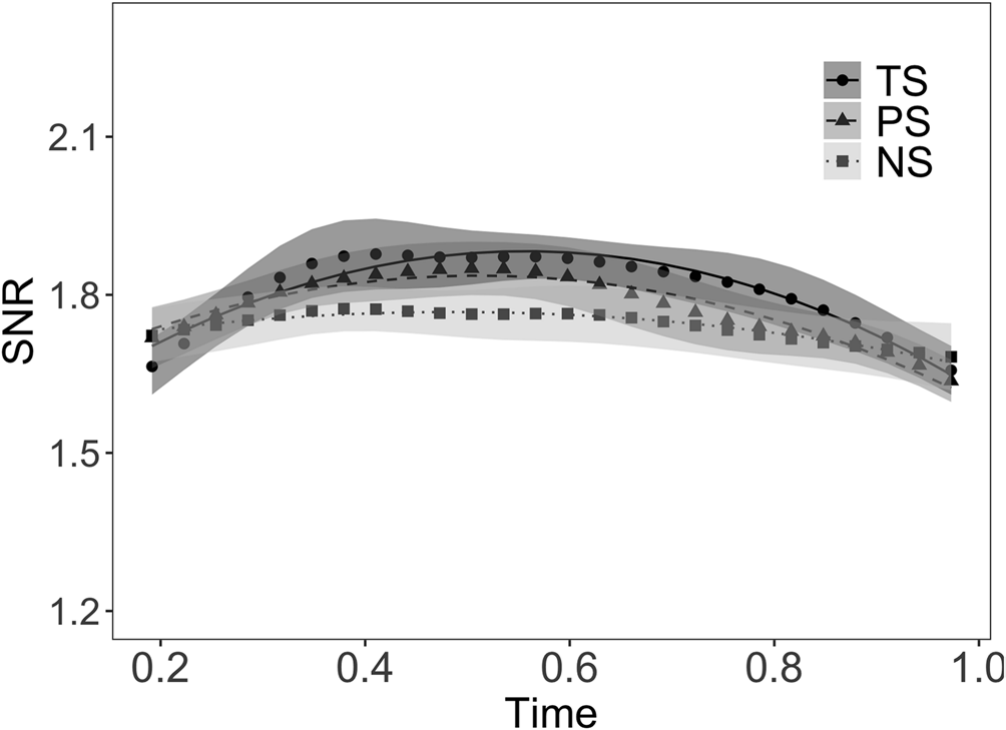
Effect of morpheme status for suffixes. The SSVEP time course from RESS component optimized for 12.50 Hz is shown. Symbols represent EEG data; shadows represent standard error of the means (SEMs) adjusted to correctly reflect the variance in the within-subject design (Morey 2008). Lines represent growth curve analysis model fit. TS = Truly suffixed, PS = Pseudo-suffixed, NS = Non-suffixed. The figure was created using R^56^ (http://www.R-project.org) package ggplot2 (https://cran.r-project.org/web/packages/ggplot2/index.html).

The effect of Morpheme Status, along with the growth curve analysis model fit in the time interval 200–1000 ms is shown in Fig. 2, and the final model parameters in Table 2.

**Table 2:** Final model parameters for suffixes.

Predictors	Estimates	Std. Error	Statistic	*p* adjusted
**SNR**				
Intercept (TS)	1.81	0.11	15.96	< 0.001
Linear	− 0.08	0.05	− 1.66	0.145
Quadratic	− 0.34	0.05	− 6.75	< 0.001
PS	− 0.03	0.01	− 2.23	0.046
NS	− 0.07	0.01	− 4.74	< 0.001
Linear * PS	− 0.09	0.07	− 1.23	0.244
Linear * NS	− 0.01	0.07	− 0.13	0.895
Quadratic * PS	0.09	0.07	1.26	0.244
Quadratic * NS	0.24	0.07	3.36	0.002
**Random effects**				
σ2	0.04			
τ00 subj	0.22			
ICC	0.84			
N subj	17			
Observations	1326			
Marginal R^2^/conditional R^2^	0.014/0.879			

Factors were dummy coded with truly suffixed as reference level, using polynomial interpolation. Note that the *b*-coefficient (estimates) represents the adjustment with respect to the reference level. *TS* truly suffixed, *PS* pseudo-suffixed, *NS* non-suffixed.

#### Effect of morpheme status

The comparison between the TS and NS conditions (solid vs. dotted line, Fig. 2) revealed a significant effect on the intercept (*b* = − 0.07, *t* = − 4.74, *p* < 0.001), with a negative β, indicating that the average response magnitude was smaller in the NS compared to the TS condition. There was no significant effect on the linear term (*b* = − 0.01, *t* = − 0.13, *p* = 0.89), indicating that the linear increase was comparable across conditions. There was a significant effect on the quadratic term (*b* = 0.24, *t* = 3.36, *p* = 0.002), with a positive β indicating a flatter curve in the NS compared to the TS condition, and therefore, a response that changed faster in the TS compared to the NS condition.

The comparison between the TS and PS conditions (solid vs. dashed line, Fig. 2) revealed a significant effect on the intercept (*b* = − 0.03, *t* = − 2.23, *p* = 0.046), with a negative β, indicating that the average response magnitude was smaller in the PS compared to the TS condition. There was no significant effect on the linear term (*b* = − 0.09, *t* = − 1.23, *p* = 0.244), with a positive β, suggesting that the linear increase was similar. There was also no significant effect on the quadratic term (*b* = 0.09, *t* = 1.26, *p* = 0.244), showing that the slopes of two trajectories were similar.

The comparison between the PS and NS conditions (dashed vs. dotted line, Fig. 2) showed that there was a significant effect on the intercept (*b* = −0.03, *t* = −2.51, *p* = 0.027), indicating that the average response magnitude was smaller in the NS compared to the PS condition. There was no significant effect on the linear term (*b* = 0.08, *t* = 1.10, *p* = 0.27), indicating that the linear increase was similar across conditions. There was a trend toward a significant effect on the quadratic term (*b* = 0.15, *t* = 2.10 *p* = 0.053), with a positive β, indicating a flatter curve in the NS compared to the PS condition, and therefore, a response that changed faster for PS than NS.

### Inter-modulation analyses

Typically, in SSVEP paradigms, neural responses are visible at the input frequency and its harmonics. However, when there is more than one stimulation frequency, it is possible to detect responses related to the sum or difference of the two input frequencies (e.g., F1 + F2 = 31.25 or F2 − F1 = 6.25), i.e., the inter-modulation (IM) terms. IM components reflects neural interaction and the integration or convergence of different processes and have been observed in low- and high-level visual processing^57^ and visual word recognition^58^. In the present data, the IM components reflect the convergence of the perceptual processing of the two flickering word parts, and therefore the perceptual integration of the two morphological units. In other words, the IM reflects the activity of neural assemblies that respond to the conjunction of the two morphological units (stem + suffix).

**Figure 3:**
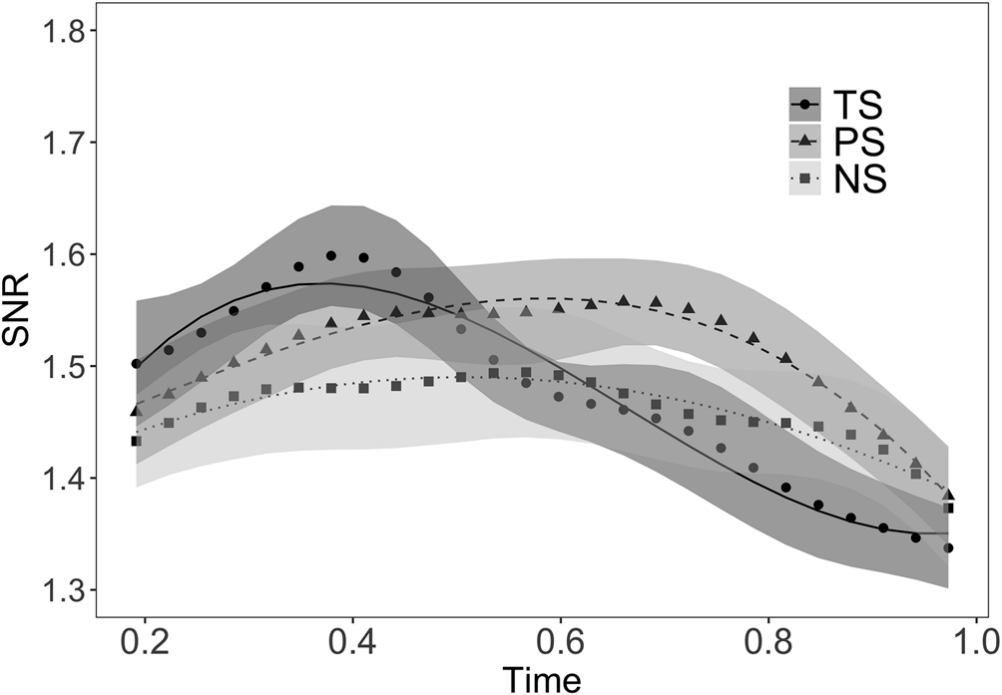
Effect of morpheme status on the 31.25 Hz inter-modulation component. The SSVEP time course from RESS component optimized for 31.25 Hz is shown. Symbols represent EEG data; shadows represent standard error of the means (SEMs) adjusted to correctly reflect the variance in the within-subject design (Morey, 2008). Lines represent growth curve analysis model fit. TS = Truly suffixed, PS = Pseudo-suffixed, NS = Non-suffixed. The figure was created using R^56^ (http://www.R-project.org) package ggplot2 (https://cran.r-project.org/web/packages/ggplot2/index.html).

We inspected the two major IM components of our setting: the sum (31.25 Hz) and the difference (6.25 Hz) of the driving frequencies. The analyses of the 6.25 Hz component revealed no significant results. The analysis of the 31.25 Hz component indicated a significantly faster response to items in the truly suffixed condition compared to the pseudo-suffixed and non-suffixed conditions on the linear term (for a detailed description of the results, see Table 3 and Fig. 3).

**Table 3:** Model parameters of the inter-modulation analyses.

Predictors	Estimates	Std. Error	Statistic	*p* adjusted
**SNR**				
Intercept (TS)	1.48	0.02	64.53	< 0.001
Linear	− 0.37	0.05	− 8.10	< 0.001
Quadratic	− 0.14	0.05	− 2.97	0.007
Cubic	0.11	0.05	2.48	0.023
PS	0.03	0.01	2.69	0.014
NS	− 0.02	0.01	− 1.28	0.218
Linear * PS	0.29	0.06	4.51	< 0.001
Linear * NS	0.29	0.06	4.47	< 0.001
Quadratic * PS	− 0.09	0.06	− 1.33	0.218
Quadratic * NS	0.02	0.06	0.27	0.784
Cubic * PS	− 0.15	0.06	− 2.32	0.031
Cubic * NS	− 0.11	0.06	− 1.71	0.116
**Random effects**				
σ2	0.04			
τ00 subj	0.20			
ICC	0.83			
N subj	17			
Observations	1326			
Marginal R^2^/conditional R^2^	0.040/0.841			

Factors were dummy coded with truly suffixed as reference level, using polynomial interpolation. Note that the *b*-coefficient (Estimates) represents the adjustment with respect to the reference level. *TS* truly suffixed, *PS* pseudo-suffixed, *NS* non-suffixed.

## Discussion

In the present study, the SSVEP paradigm was used to dissociate participants’ EEG responses to embedded stems and affixes. Three different types of target words were presented to proficient readers in a delayed naming task: truly suffixed words (*farmer*), pseudo-suffixed words (*corner*), and non-suffixed words (*cashew*). To test for neurophysiological markers of stem and affix processing in this task, stems and affixes were flickered at two different frequencies (18.75 Hz and 12.50 Hz, respectively). To examine entrainment to the stimulation frequencies, we used rhythmic entrainment source separation (RESS)^59^. A spatial filter was applied to increase spectral specificity and maximize the signal-to-noise ratio (SNR) of the steady-state response at each frequency (18.75 Hz and 12.50 Hz). The resulting SSVEPs, computed as SNR of the RESS filtered power (representing a weighted combination of all electrodes), revealed a clearly visible peak in spectral power at 18.75 Hz for stem morphemes (Fig. 5, right panel), and at 12.50 Hz for suffixes (Fig. 6, right panel).

The highest SSVEP responses were observed in posterior scalp locations as shown in the topographical maps of the frequency-specific spatial filter (left panels of Figs. 5 and 6). The observed pattern is consistent with the notion that the occipital and parietal lobes are major contributors to the generation of SSVEPs^60^, but also suggest that our responses reflected the activity of rather low-level stages of processing.

Indeed, the maps associated with stem morphemes (Fig. 5, left panel) and suffixes (Fig. 6, left panel) revealed clearly distinct topographic differences that are fairly consistent with the lateralization of the visual system^61^. The maps for stems showed that the highest recorded response was distributed over the right visual posterior cortex. In contrast, the maps for suffixes showed a wider distribution that was centred over the left visual posterior cortex. Since target words were presented centrally, stem morphemes appeared slightly to the left of the centre of the screen, in the left visual field, and were presumably processed initially by the right visual cortex, while suffixes occurred slightly to the right of the centre of the screen, in the right visual field, and were therefore initially processed by the left visual cortex. Therefore, these topographical differences could at least partially be due to the lateralisation of the visual system. It is worth noting that consistent with our findings, a right-lateral effect of morphological complexity has been previously reported in the M170 component, localized in the posterior inferior region^19^.

Another notable difference between the topographic maps of stems and suffixes is that SSVEP activity was more widespread for suffixes than for stems. One possibility is that suffixes elicit a more diffuse response because they have a more generalizable, complex meaning compared to stems. Another possibility is that the observed differences in SSVEP scalp distribution were due to the different input frequencies of stems and suffixes, with different input frequencies eliciting peaks at different electrode locations^60^. Alpha rhythm has been shown to be the prominent rhythm in the visual cortex, where visual stimuli can elicit a SSVEP response that peaks exactly at 12.50 Hz^62^. Thus, the stimulation frequency that we used for suffixes (12.50 Hz) may have matched the natural frequency of processing of this kind of stimuli producing a stronger cortical resonance effect. Whether or not differences in the spread of SSVEP activity between stem and suffixes reflect selective engagement of specific processing networks for stems and suffixes or are a reflection of stimulus frequency, or both, is unclear, and goes beyond the scope of the present study. Future research should examine more systematically topographic differences with different SSVEP input frequencies. The following discussion will focus on amplitude changes in SSVEP activity between conditions (TS, PS, NS), rather than the spatial localisation of the SSVEP signal.

To address the question of whether or not SSVEP responses are sensitive to the morphemic status of embedded words, we compared two conditions in which the embedded word formed part of an exhaustively decomposable letter string (as was the case in the truly suffixed and pseudo-suffixed conditions) to a condition in which the embedded word was an entirely non-morphological letter string (as was the case in the non-suffixed control condition). Based on evidence showing that skilled readers are experts at rapidly decomposing morphologically complex words (*farm* + *er*) and pseudo-complex words (*corn* + *er*) into morphemic subunits^2,3^, we had hypothesised that stems embedded in truly and pseudo-suffixed words should elicit clearly distinct pattern of SSVEP activity compared to words embedded in non-suffixed words (e.g., the *cash* in *cashew*). Our results confirm this hypothesis, by showing that the time course of the SSVEPs was modulated by morphemic status, as demonstrated by the polynomial terms analysis, with the three trajectories showing different activation pattern across time (Fig. 1).

The temporal dynamics of SSVEPs reflect the efficiency of the neural assembly recruited by the stimulus to synchronize its response to the flickering event^46–48^. For example, it is well established that in paradigms in which attention is manipulated, attended items systematically exhibit greater amplitudes compared to unattended items^41^. With respect to visual word recognition, it has been previously reported that words elicit larger SSVEPs than pseudowords^43^, high-frequency words larger SSVEPs than low-frequency words^43^, and congruent syllables larger SSVEPs than incongruent syllables^44^. Here, we extend this prior work to the context of morphologically complex words, showing that embedded stems (both real stems and pseudo-stems) elicit a peak in SSVEPs, at about 400 ms (Fig. 1), which occurred about 200 ms earlier than in the non-suffixed condition. SSVEP responses to truly suffixed and pseudo-suffixed stems changed faster than responses to words embedded in non-suffixed words. Indeed, the maximum response peak in the non-suffixed condition was reached around 600 ms. The shorter SSVEP latencies for (pseudo)stems suggest that stem processing was facilitated by increased information processing speed compared to stimuli in the non-suffixed condition.

Our results thus shed new light on the neural underpinnings of morphological processing in the human brain. The clearly distinct stem-effects for truly and pseudo-suffixed words on the one hand, and non-suffixed words on the other hand, are in line with prior EEG research, showing that ERPs to truly suffixed and pseudo-suffixed words typically pattern together during the early time windows^10,12,14^. Our research takes these findings one step further by using the SSVEP paradigm to pinpoint neural responses that are specifically tuned to the embedded morphemic units. The earlier peak in participants’ SSVEPs to stems embedded in truly suffixed words (*farm* in *farmer*) and pseudo-suffixed words (*corn* in *corner*) speaks in favour of a mechanism by which skilled readers rapidly decompose complex words into morphemic subunits. These findings fit with the idea that the decomposition of complex words is a purely structural, form-based chunking mechanism, a feature which forms part of several theoretical models of morphological processing^3,6,22,25,29,32,34^. Following morphemic segmentation, the embedded (pseudo)stem is rapidly activated in the orthographic lexicon, thus yielding a faster synchronization to the stimulus rhythm. In contrast, morphemic segmentation is not successful for non-suffixed words, thus explaining why SSVEPs to embedded words in this condition (*cash* in *cashew*) emerged later than in the two suffixed conditions.

An additional goal of the current study was to address the question of whether or not SSVEP responses are sensitive to the morphemic status of embedded suffixes. Similar to the analyses of the stem-effects, the critical comparison in the suffix data was the contrast between the two suffixed conditions and the non-suffixed condition in which the embedded word was accompanied by a non-morphemic ending (*ew* in *cashew*). Since suffixes always occur in combination with a stem morpheme and are never encountered as free-standing units, it is experimentally more difficult to investigate the specific role of the suffix in participants’ reading behaviour. Although a number of previous masked primed lexical decision studies^63,64^ have reported facilitatory priming effects for prime-target pairs sharing the same suffix (e.g. *baker-WALKER*), these effects were calculated on the basis of lexical decision responses to complex whole words, making it difficult to derive conclusions concerning suffix-specific activation thresholds. The affix frequency modulation of the M170 component provided some initial evidence for the idea that affixes are associated with distinct neurophysiological activation pattern^20^. In the present study, we were able to measure specific neural responses to suffixes that were naturally embedded in whole words, using frequency tagging, without having to pursue the less natural option of presenting suffixes in isolation.

The results revealed that the average magnitude of SSVEP responses to true suffixes (*er* in *farmer*) were larger than to pseudo-suffixes (*er* in *corner*) and non-suffixes (*ew* in *cashew*; Fig. 2). Moreover, the SSVEP magnitude was larger for pseudo-suffixes than non-suffixes. These findings suggest that the size of the recruited neuronal assembly was largest for true suffixes, average for pseudo-suffixes, and smallest for non-suffixes. The larger response for suffixes in the truly suffixed condition compared to the pseudo-suffixed and non-suffixed conditions suggests that the activation of suffixes benefitted from the semantically transparent context in which they occurred (e.g., *farmer*: ‘someone who farms’). In contrast, the suffixes of pseudo-suffixed words and the non-morphemic endings in the non-suffixed condition did not constitute a semantically plausible letter sequence. Moreover, the polynomial analyses revealed that, although the overall linear increase was broadly similar across conditions, the quadratic terms indicated faster SSVEPs changes for true suffixes and pseudo-suffixes compared to non-suffixes, and faster SSVEPs changes for pseudo-suffixes compared to non-suffixes. The clearly distinct SSVEP responses in the pseudo-suffixed and non-suffixed conditions, in the form of faster power changes and a more consistent increase across time, show that even in the absence of semantics, pseudo-suffixes yielded a neural activation pattern that was dissociable from simple non-morphemic letter sequences.

The current data suggest that the activation of suffix representations is reliant on a combination of two different mechanisms: morphological decomposition (also evidenced in the stem data) and semantic feedback. The activation of affix representations in the orthographic lexicon explains the faster power changes in the two suffixed conditions compared to the non-suffixed condition. In addition, the suffixes of words with a genuine morphological structure that are active in the orthographic lexicon benefitted from top-down feedback connections from the level of semantic representations^33,34^, thus leading to an increased SSVEP response in this condition.

Why is it the case that semantic context is more important for suffix than for stem processing? Since suffixes are bound morphemes (i.e., never occur in isolation), the reading system relies on the context in which a suffix occurs to identify if it acts as a real morpheme or a pseudo-morpheme. At the level of the orthographic lexicon, the reading system does not distinguish between a real suffix (*er* in *farmer*) and a pseudo-suffix (*er* in *corner*), because both form part of a letter string that is exhaustively decomposable into stem + suffix. It is only at the level of semantics where real suffixes and pseudo-suffixes become clearly dissociable. Indeed, the inter-modulation results support the notion that the interaction between the neural responses to two semantically congruent morphemic units (e.g., the stem *farm* and the suffix *-er*), leads to faster visual word processing as was evidenced by the IM trajectory of the truly suffixed condition (see Fig. 3). In contrast to suffixes, stems elicit more robust orthographic form representations that are less affected by semantic feedback, presumably because the majority of stems are free morphemes (i.e., also occur in isolation). Indeed, Beyersmann and Grainger^33^ argue that affixes, but not stems (with the exception of bound stems, e.g. *flate* in *deflate*), are marked as ‘bound’ at the level of the orthographic lexicon, which sends an early signal to the reading system that the lexico-semantic context is needed to complete the evaluation of the input letter string. This thus provides an explanation for why the present SSVEP data revealed a semantic activation boost for suffixes, but not for stems.

The present study highlights the sensitivity of the SSVEP paradigm to embedded morphemic reading units. Building on previous SSVEP research, our findings suggest that important functional reading units, such as words, morphemes and syllables, are characterized by more coherent neural dynamics and larger networks, including stronger associations with sensorimotor areas and higher-order regions. The stem data revealed an earlier SSVEP peak in both the truly suffixed and pseudo-suffixed conditions, thus providing evidence for the semantic-independent activation of embedded stems during reading. The suffix data also showed a dissociation between the SSVEP response to suffixes compared to non-suffixes, but an additional activation boost was observed in the truly suffixed condition, suggesting that true suffixes were privileged, presumably due to their semantic transparency. These SSVEP data are consistent with the hypothesis that the neurophysiological underpinnings of stem and affix processing are clearly dissociable.

## Methods

### Participants

Seventeen proficient readers, naive to the purpose of the experiment, participated in the experiment as paid volunteers (age 19–36 years, mean = 22). The number of participants corresponded to previous work^43^, who found robust frequency and lexicality effects using the same task and SSVEP paradigm. All participants were native French speakers, they had normal or corrected-to-normal vision and they had no history of developmental disorders. Participants gave informed written consent prior to participating in the experiment. The procedure was approved by the local ethics committee of the University of Aix-Marseille. The experiment was performed in accordance with relevant guidelines and regulations and in accordance with the Declaration of Helsinki.

### Apparatus and stimuli

Participants were seated 56 cm from the monitor in a dimly-lit, electrically isolated room. Stimuli were generated using OpenSesame 3.0^65^ and PsychoPy^66^ and presented on a 17-in. cathode ray tube monitor (resolution 800 × 600 pixels, refresh rate 75 Hz).

We used 153 French words selected from the Lexique database^67^, which were identical to the ones previously used in a lexical decision task^68^. The 153 words included 51 French pseudo-suffixed target words (e.g., *couette* [engl. *blanket*], consisting of the pseudo-stem *cou* [engl. *neck*] and the pseudo-affix -*ette*). Pseudo-suffixed target words were selected such that the whole word never shared any semantic relationship with the embedded pseudo-stem. In addition, we used 51 truly suffixed target words (e.g., *pochette* [engl. *little pocket*], consisting of the stem *poche* [engl. *pocket*] and the affix -*ette*), where whole word and stem always clearly shared a semantic relationship. Truly suffixed (TS) and pseudo-suffixed (PS) target words shared exactly the same suffixes. Finally, a third set of 51 non-suffixed (NS) items was added (e.g., *principe* [engl. *principal*]), which consisted of an embedded word (*prince* [engl. *prince*]) and a meaningless non-morphemic ending (-*ipe*). The experimental design involving the key comparison between truly suffixed, pseudo-suffixed and non-suffixed words was based on a widely replicated morphological processing methodology^2,3^. Although this design standardly involves a larger proportion of (pseudo)affixed than non-affixed items, which is necessary in order to have an equal number of items per condition, it has been shown that complex word reading is not affected by the proportion of affixed trials in the task^69^. Participants were asked to read each item aloud, which required the processing of whole letter strings (i.e., suffix detection was not sufficient to complete the task). All target words were nouns. The three sets of targets words were matched on written word frequency, spoken word frequency, number of letters, number of phonemes, number of syllables, orthographic neighborhood, phonological neighborhood, ending length and uniqueness point^68^.

### Procedure

**Figure 4:**
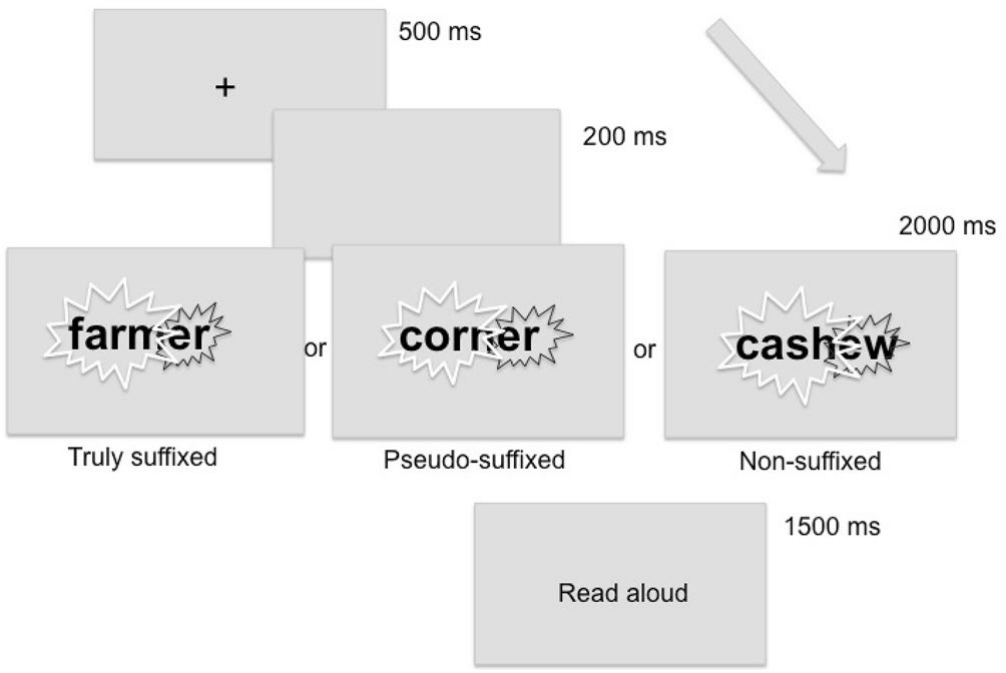
Delayed naming task procedure. The first part of the word, matching the embedded word (e.g., *farm, corn, cash*), flickered on and off at 18.75 Hz. The remaining part of the word (e.g., *er, er, ew*) flickered on and off at 12.50 Hz. The figure was created using Microsoft PowerPoint (https://www.microsoft.com/).

Participants performed a delaying naming task (Fig. 4). Words were presented in black against a white background, at the centre of the screen, using Selectric font. Each trial started with a fixation point (black cross on a white screen) displayed at the center of the screen for 500 ms. After an empty screen lasting 250 ms, the flickering word was presented for 2000 ms. Then, a screen prompted participants to read the word aloud. The next trial started after 4000 ms. After 2000 ms, an empty screen replaced the response screen. The interval from the start of one trial to the start of the next trial was about 7500 ms.

To track the responses to the two different segments of the word (i.e., stem and suffix), we used frequency tagging of stimulus to evoke SSVEPs separately for the stem and the suffix. The first part of the word, matching the word stem, flickered on and off at 18.75 Hz (4 monitor refresh cycles per period: 1 on- and 3 off-cycles). The remaining part of the word, matching the suffix, flickered on and off at 12.50 Hz (6 monitor refresh cycles per period: 1 on- and 5 off-cycles).

Vocal responses were assessed online by the experimenter in order to be sure the participant was engaged in the task. Due to the long presentation time, accuracy was 100%. Each session consisted of three blocks of 51 trials each with a brief pause between each block. The block sequence was counterbalanced between participants while the order of trials within each block was randomized for each subject.

### EEG recording and preprocessing

EEG activity was acquired at 512 Hz using a Biosemi Active Two system provided with 64 Ag/AgCl sintered active electrodes. Electrodes were mounted on an elastic cap (Electro-Cap, Inc. Eaton, OH) that was positioned according to the 10–20 International system (American Clinical Neurophysiology Society, 2006). Two additional electrodes (CMS/DRL) were used as on-line reference (see www.biosemi.com). Three extra electrodes were used to monitor eye movements and blinks (two placed at the outer canthi of the eyes, one placed below the left eye). Other two extra electrodes were used for an off-line re-referencing (placed behind ears on mastoid bone).

For the EEG analysis, we used EEGLAB^70^, ERPLAB^71^ and Fieldtrip^72^ toolboxes for Matlab and Matlab customized functions (Matlab 2014, The Mathworks). Data were re-referenced off-line to the average of left and right mastoid electrodes, bandpass filtered from 5 to 100 Hz and then segmented to include 200 ms before and 2000 ms after stimulus onset. Epoched data were normalized based on a prestimulus period of 200 ms, and then evaluated according to a sample-by-sample procedure to remove noisy sensors that were replaced using spherical splines. Likewise, EEG epochs that contained data samples exceeding threshold (100 uV) were excluded on a sensor-by-sensor basis, including horizontal and vertical eye channels. On average, 1.28% of the data were interpolated and 3.77% of the data rejected. In addition, ten items (6.29% of the data) were removed, because the embedded stem (e.g., *calc* in *calcaire*) did not correspond to the orthographic form of the corresponding real word (e.g., *calque*). The 10 removed items included 1 non-suffixed, 4 truly suffixed, and 5 pseudo-suffixed items. We ensured that the three sets of targets words were still matched following item removal on written word frequency, spoken word frequency, number of letters, number of phonemes, number of syllables, orthographic neighborhood, phonological neighborhood, ending length and uniqueness point. Slow drifts were removed to reduce “sawtooth” artifacts in the Fourier spectrum^73^.

### SSVEP extraction

To examine the time course of the SSVEP response, the data were segmented to include 200 ms before and 2000 ms after word onset and then subjected to a short-time Fourier transformation with a moving hanning window of 800 ms in steps of 32 ms. We applied rhythmic entrainment source separation (RESS)^59^ to extract SSVEPs from the ongoing spontaneous oscillatory activity. RESS offers several advantages over more traditional ways of analyzing SSVEPs. The procedure broadly consists in the computation of a spatial filter that maximizes the explained variance for the frequency-specific SSVEP signal relative to the broadband ongoing electrophysiological activity. The RESS takes weighted combinations of all electrodes to produce a single time series, i.e., the RESS component, therefore, allowing to bypass the stage of subjective electrode selection. In addition, the RESS boosts the signal-to-noise ratio (SNR) of SSVEPs that can be difficult to isolate from the ongoing activity in cognitive paradigms that require short presentation time.

**Figure 5:**
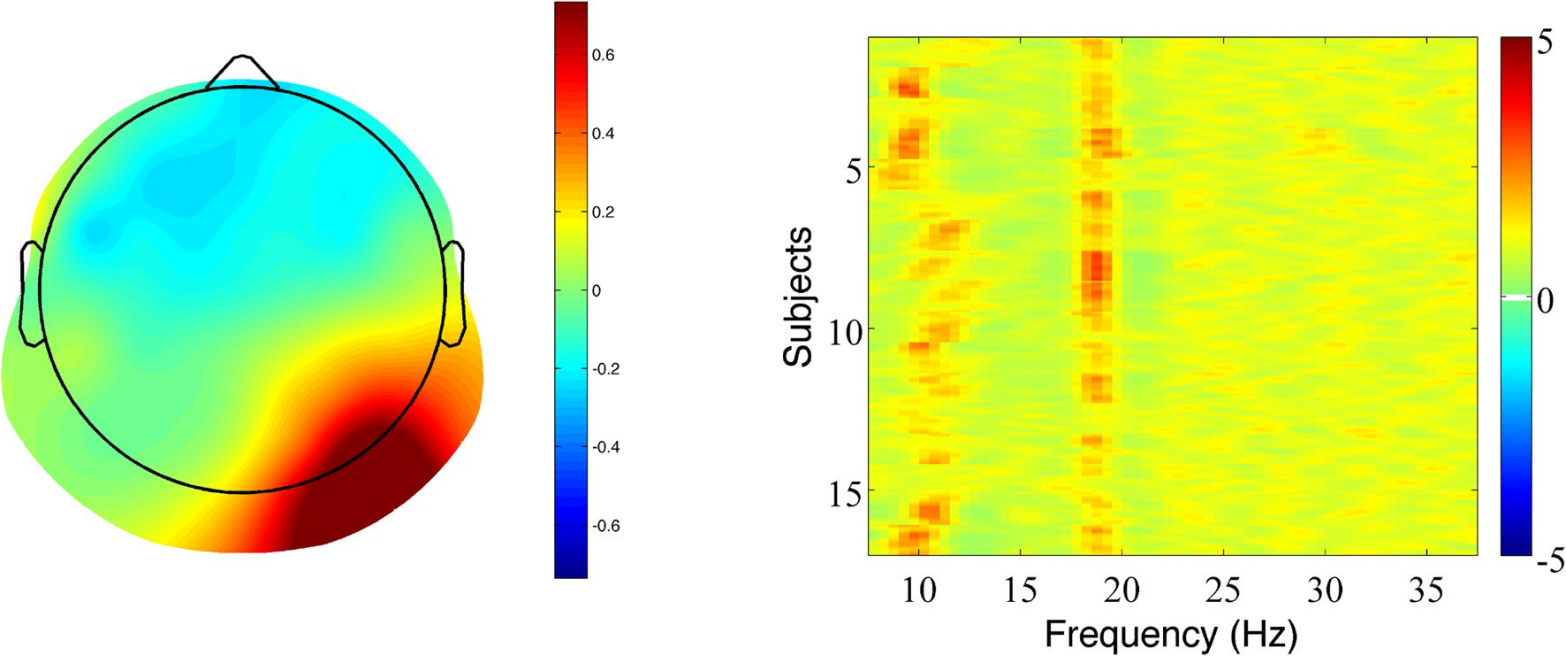
Rhythmic entrainment source separation (RESS) filtered power spectra optimized for 18.75 Hz (Stem). Left panel: Topographic map, averaged across participants. Topoplot color was normalized to [− 1 1] to facilitate visual comparison. Right panel: Spectral density (SNR) between 5 and 37 Hz for each participant (rows). The vertical red line at 18.75 Hz corresponds to the entrainment at the stimulation frequency. The left panel of this figure was created using FieldTrip (https://www.fieldtriptoolbox.org/)^72^, the right panel using MATLAB (https://www.mathworks.com/products/matlab.html)).

**Figure 6:**
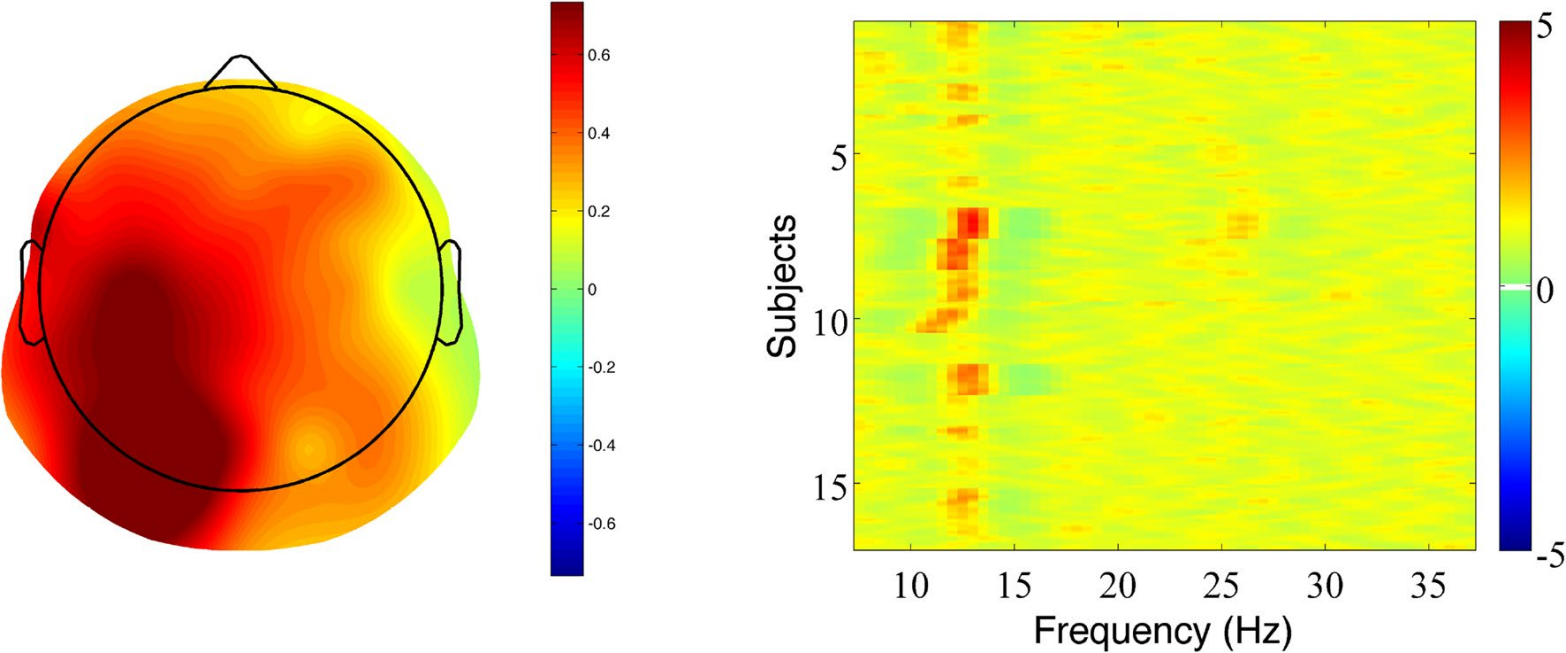
Rhythmic entrainment source separation (RESS) filtered power spectra optimized for 12.50 Hz (Suffix). Left panel: Topographic map, averaged across participants. Topoplot color was normalized to [− 1 1] to facilitate visual comparison. Right panel: Spectral density (SNR) between 5 and 37 Hz for each participant (rows). The vertical red line at 12.50 Hz corresponds to the entrainment at the stimulation frequency. Alpha activity is not visible in the right panel, likely for two reasons. First, the SSVEPs are calculated as SNR (i.e., the difference between power at the stimulation frequency and power at the average of neighbouring frequencies). Thus, the alpha band activity and the activity at 12.50 Hz are partially cancelling each other out. Second, the spatial locations most responsive to 12.50 Hz are more diffuse than for the 18.75 Hz input. For the latter (see left panel of Fig. 5) the most active locations were confined to the occipital area, i.e., the visual cortex, where alpha activity is the prominent frequency. On the contrary, the spatial locations for 12.50 Hz input were more diffuse and included parietal/central areas where alpha activity tends to be less prominent. The left panel of this figure was created using FieldTrip (https://www.fieldtriptoolbox.org/)^72^, the right panel using MATLAB (https://www.mathworks.com/products/matlab.html).

Two spatial filters were computed separately for each participant (i.e., one filter for 18.75 Hz and another for 12.50 Hz) by pooling data across all three conditions (i.e., TS, PS and NS), to avoid overfitting. The two filters were computed over the window [0–1000] to restrict the analysis to the spatial locations most active during the early stages of visual word recognition (see also below). The analysis was restricted to the fundamental frequency (e.g., F = 18.75 Hz) rather than higher harmonics (i.e., integer multiples of the fundamental frequency F, such as F = 37.5 Hz) because the response was clearly stronger at that frequency. To facilitate comparisons across subjects, SSVEPs were computed as SNRs, that is the ratio between RESS filtered power at the stimulation frequency to power at the average neighbouring frequencies (± 2.5 Hz, excluding ± 1 Hz around the stimulation frequency). The RESS topographical maps (RESS spatial filters) for the two input frequencies used in the experiment, i.e., 18.75 Hz and 12.50 Hz, are shown in the left panels of Figs. 5 and 6.

### SSVEP entrainment

For the purpose of showing the entrainment to the stimulation frequencies, we computed the Fourier transform over the 0–1000 ms time window. The SNR for each participant, filtered with the RESS optimized for 18.75 Hz, are shown in Fig. 5 (right panel). The spectral response showed a clear peak at the stimulation frequency for the stem (red vertical line at 18.75), as well as increased activity around the alpha band. Alpha band activity at approximately 10 Hz tends to be the dominant frequency in the awake brain^74^, and in particular in the visual cortex. In the present study, alpha activity was likely related to the relaxed state of the participants and low-task demands. Concerning the suffix-tagging at a stimulation frequency of 12.50 Hz, the SNR for each participant filtered with the RESS optimized for 12.50 Hz are shown in Fig. 6 (right panel), which clearly shows a peak (vertical red column) at the stimulation frequency.

### Polynomial interpolation

We modelled the time course of the effects using polynomial interpolation, a method that has been developed in the context of growth curve analyses^50^ and is well suited to model change over time. Because of the complex oscillatory nature of the signal and the nonlinear trajectory of the responses over time, the response patterns were not adequately described by straight lines (i.e., linear regression) but rather by curvilinear shapes with multiple turning points (i.e., local trajectory extremes, positive or negative peaks). Among the families of functional forms (i.e., shapes used to describe the data) able to fit curvilinear patterns, higher-order polynomial functions offer numerous advantages: facility of implementation, dynamic consistency and great accuracy^50^.

**Figure 7:**
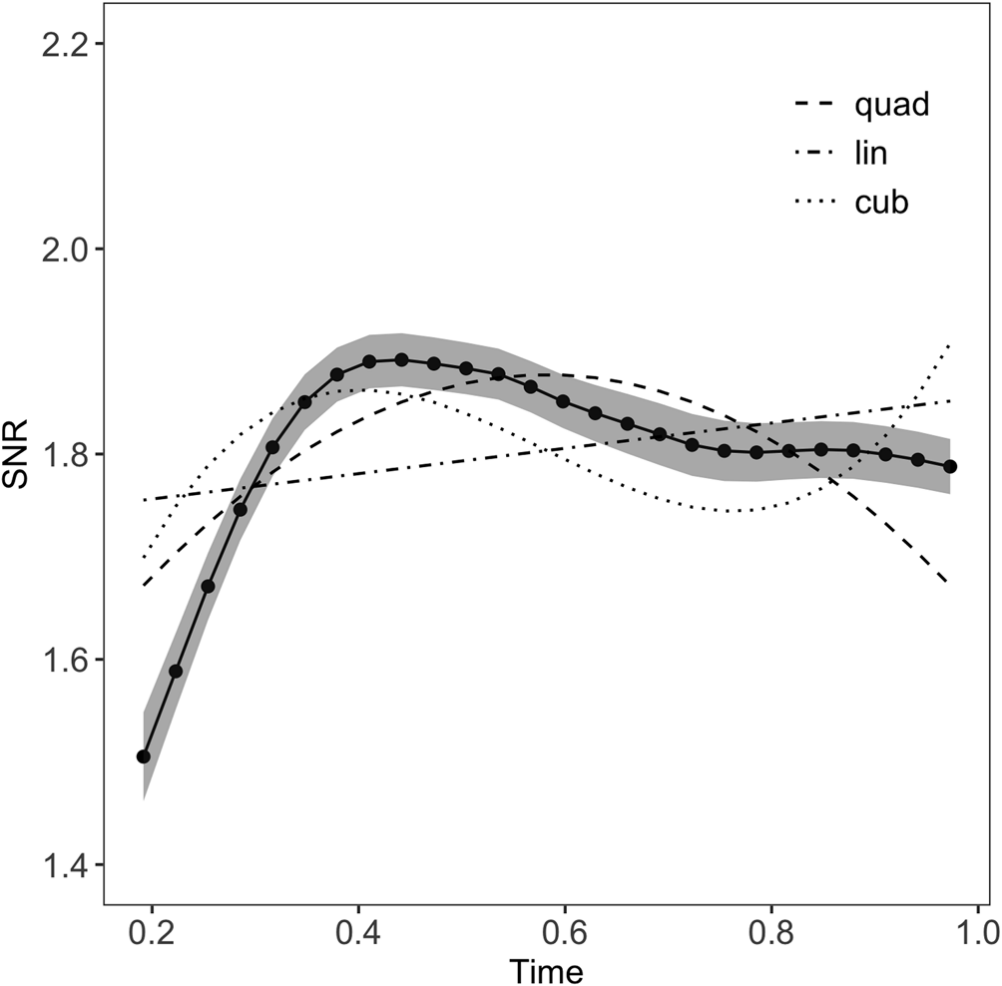
Change in the SSVEP trajectory, as captured by the polynomial terms. The plot shows the data (black dots and solid line) and the fitting of three different increasingly complex models. The first model (dot-dashed) included just the linear term. The second model (dashed) included the linear and the quadratic terms. The last model (dotted) included all three polynomial terms: linear, quadratic, and cubic. The figure was created using R^56^ (http://www.R-project.org) package ggplot2 (https://cran.r-project.org/web/packages/ggplot2/index.html).

In natural polynomials, individual time terms tend to be correlated, undermining the stability of parameter estimates and the independency of their evaluation. Therefore, orthogonal polynomials were used, by centring the polynomials to the selected time interval^50^. As a result, the intercept did not correspond to the value at time zero but to the overall average across the selected interval (200–1000 ms from word onset). The analyses focussed on the earlier time window rather than the entire 2000 ms time window, because prior EEG studies have shown that the processing of embedded reading units, including syllable processing^44,75^ and morpheme processing^10,12,14^, are best captured in the early stages of visual word recognition. The polynomial terms were calculated, including a linear, quadratic, and cubic term, to capture the change in the SSVEP trajectory (Fig. 7). The β coefficient of each of the three polynomial terms represented a distinct modulation of the change in response trajectory. The coefficient of the linear term corresponded to the average rate of change across the time interval. The coefficient of the quadratic term represented the sharpness of the central curve peak. Therefore, this coefficient tracked changes occurring at a pace that was different from the average (the higher the absolute value of the coefficient, the higher the pace). A negative quadratic coefficient was represented by a downward parabola, a trajectory that after raising to the local maximum (i.e., positive peak) showed a decrement of magnitude. The cubic term reflected the presence of a point of inflexion (i.e., a change of concavity, from concave downward to concave upward or vice versa), with its coefficient representing the sharpness of the two peaks (local maximum and local minimum).
